# Supplementary material for: Non-loss engraved circuit patterning method of semi-liquid metal for precision recyclable multi-substrate circuits
Source: Nat Commun. 2025 Nov 27;16:11690. doi: 10.1038/s41467-025-66815-4 (PMC12748806; doi:10.1038/s41467-025-66815-4)
Supplement: Supplementary file 1 — Supplementary Information [file 41467_2025_66815_MOESM1_ESM.pdf]

Non-loss Engraved Circuit Patterning Method of Semi-liquid Metal for Precision Recyclable Multi-substrate Circuits

Xiaoqing Li[1,2], Tianyu Li[2], Yubing Liu[1], Chengjie Jiang[2], Yiyi Chen[2], Hui Zong[2], Zihang Zhang[2], Jianye Gao[3], Jing Liu[1]*, Rui Guo[1,2]*

1 State Key Laboratory of Cryogenic Science and Technology, Technical Institute of Physics and Chemistry, Chinese Academy of Sciences, Beijing 100190, China

2 School of Precision Instrument and Opto-Electronics Engineering, Tianjin University, Tianjin 300072, China

3 Department of Biomedical Engineering, School of Medicine, Tsinghua University, Beijing 100084, China

* Correspondence to:

Jing Liu, State Key Laboratory of Cryogenic Science and Technology, Technical Institute of Physics and Chemistry, Chinese Academy of Sciences, Beijing 100190, China. Email: jliu@mail.ipc.ac.cn

Rui Guo, State Key Laboratory of Cryogenic Science and Technology, Technical Institute of Physics and Chemistry, Chinese Academy of Sciences, Beijing 100190, China. Email: guorui@mail.ipc.ac.cn

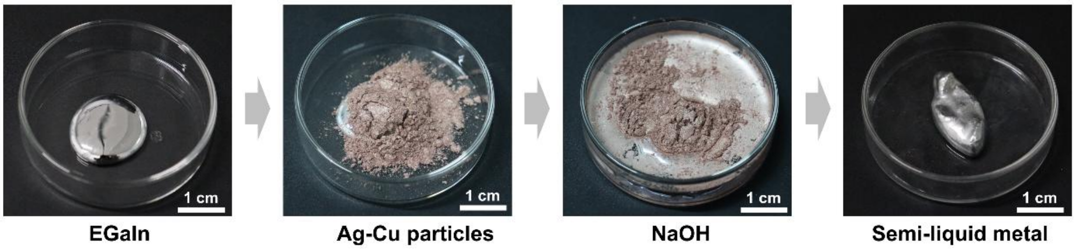

Supplementary Fig. 1 Photos of the preparation process of semi-liquid metal.

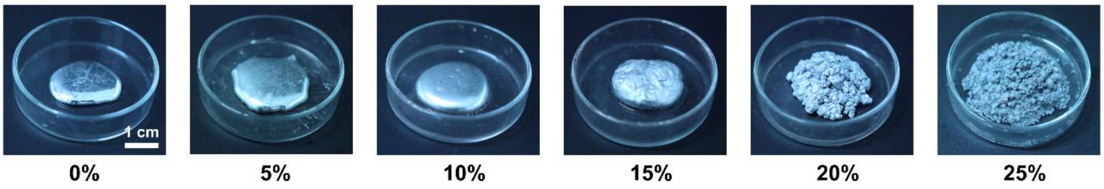

Supplementary Fig. 2 Photos of semi-liquid metals with different doping ratios.

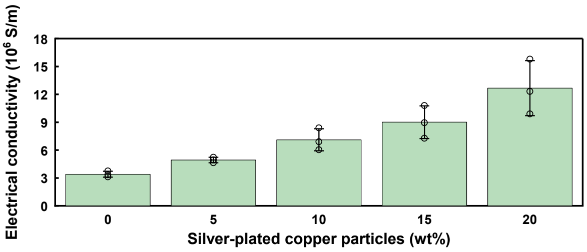

Supplementary Fig. 3 The electrical conductivities of semi-liquid metal with varying doping ratios of silver coated copper particles. Data are represented as mean ± s.d. n = 3 samples.

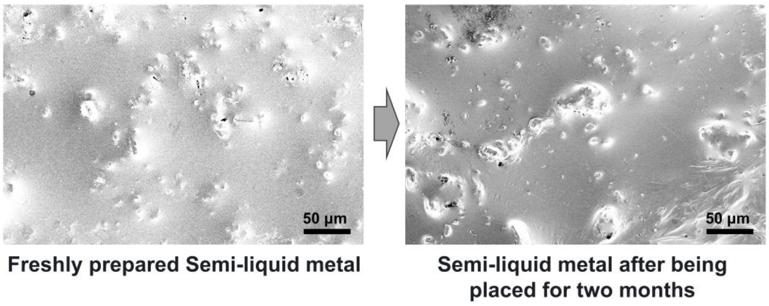

Supplementary Fig. 4 SEM images of the freshly prepared semi-liquid metal and that has been stored for two months (3 biological replicates).

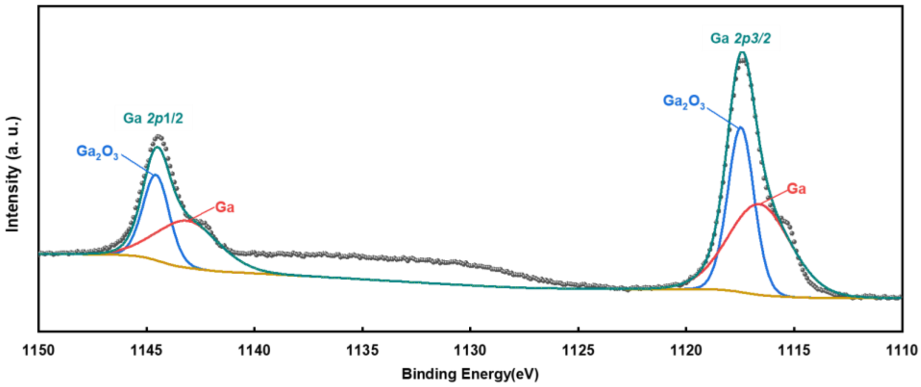

Supplementary Fig. 5 XPS curves of the oxide film on the surface of semi-liquid metal.

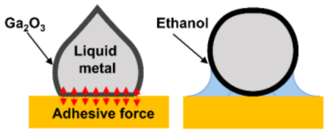

Supplementary Fig. 6 Schematic diagram of the formation mechanism for the difference in contact angle of liquid metal droplets between air and ethanol.

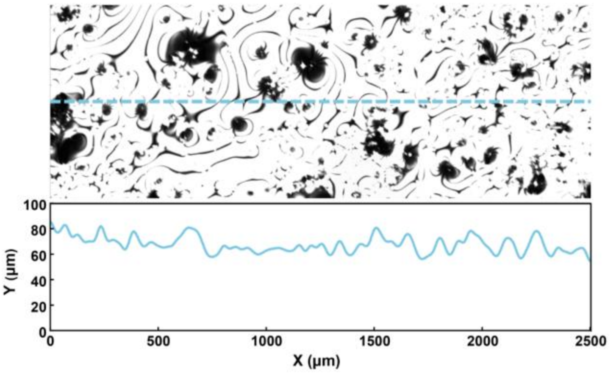

Supplementary Fig. 7 The contour curve of the semi-liquid metal film (3 biological replicates).

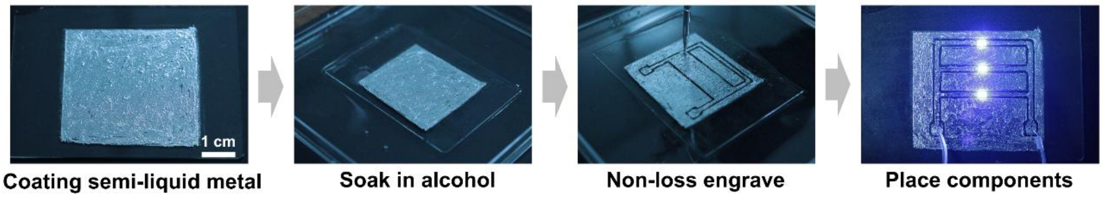

Supplementary Fig. 8 Photos of the operation process of the non-loss engraved pattern method.

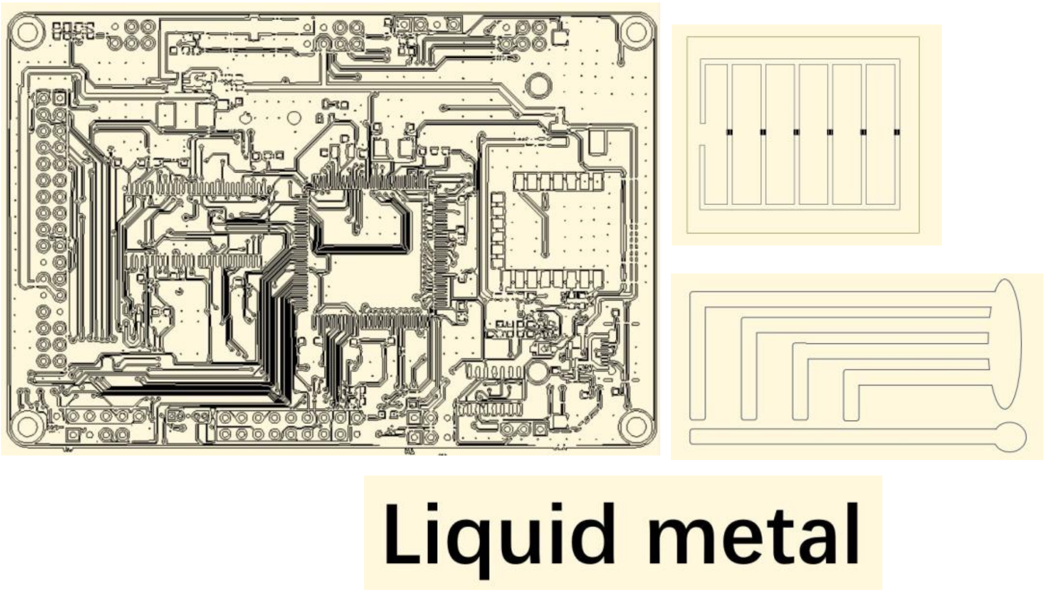

Supplementary Fig. 9 Needle tip moving paths for circuit patterns demonstrating multiple advantages of the non-loss engraved pattern method.

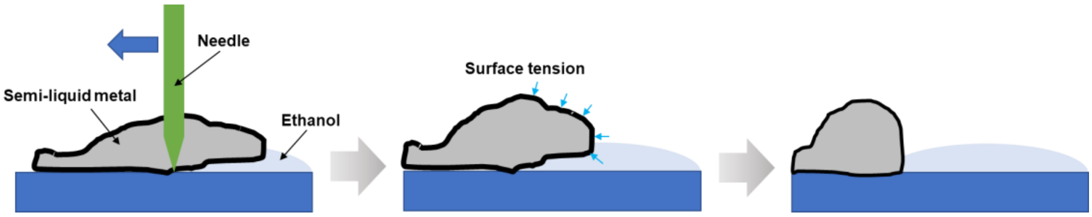

Supplementary Fig. 10 Schematic illustration of the mechanism by which alcohol prevents semi-liquid metal from re-adhering to the substrate.

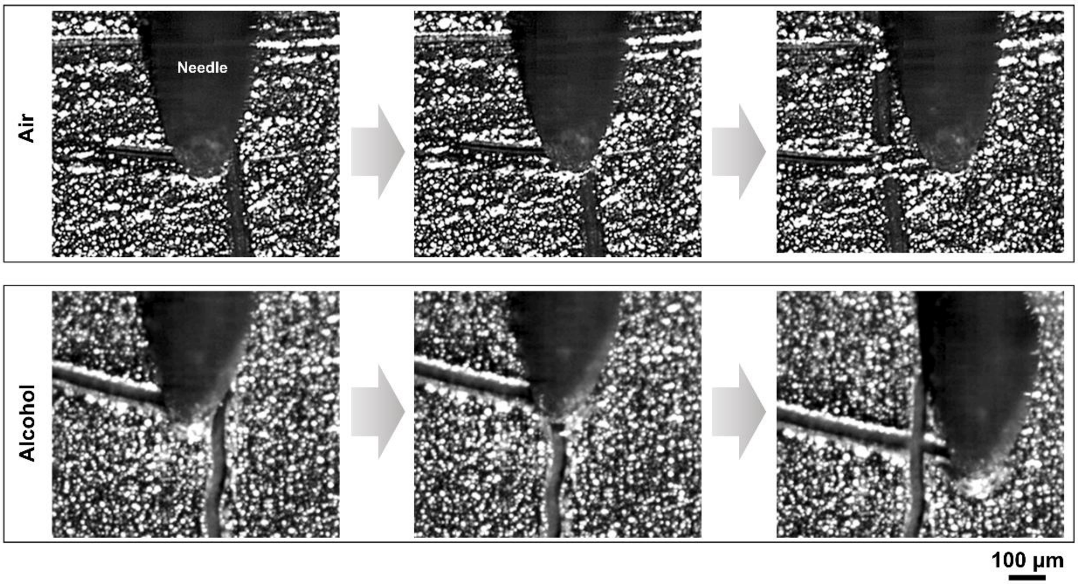

Supplementary Fig. 11 High-speed camera images of needle tip scratching cross-shaped marks in air and alcohol (3 biological replicates).

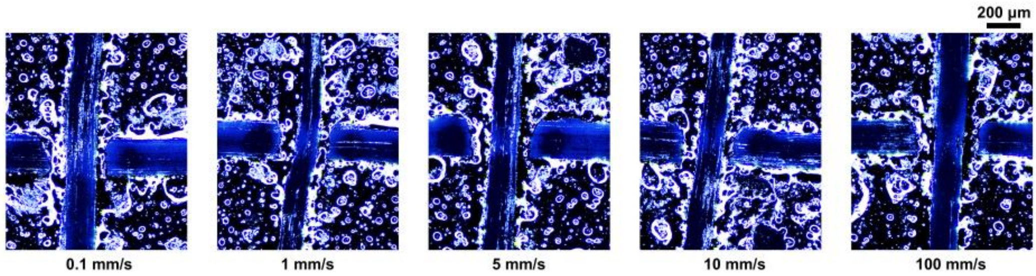

Supplementary Fig. 12 The adhesion effect of the semi-liquid metal caused by the needle tip on the cross-scratch area under different moving speeds (3 biological replicates).

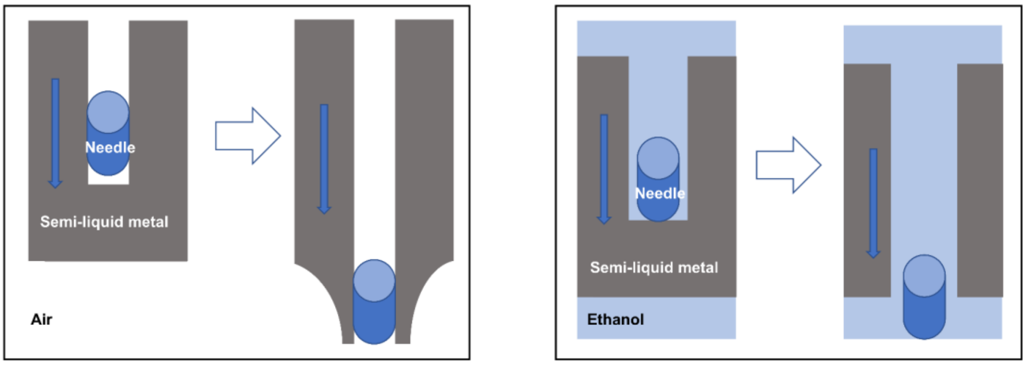

Supplementary Fig. 13 Schematic diagram of the mechanism by which ethanol prevents semi-liquid metal adhesion at secondary scratch.

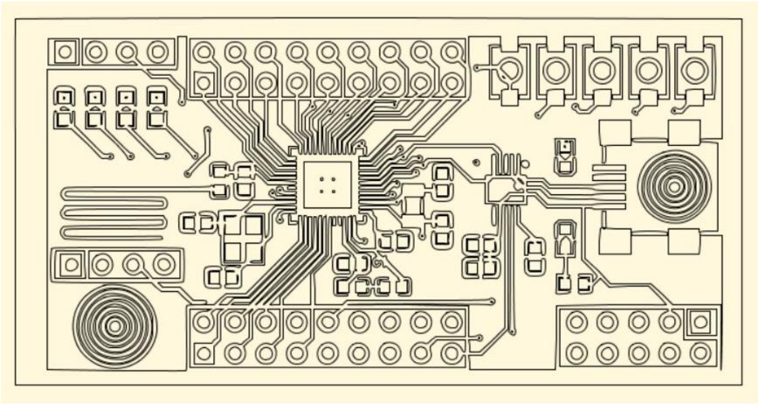

Supplementary Fig. 14 Needle tip moving paths for complex semi-liquid metal patterns on glass plates.

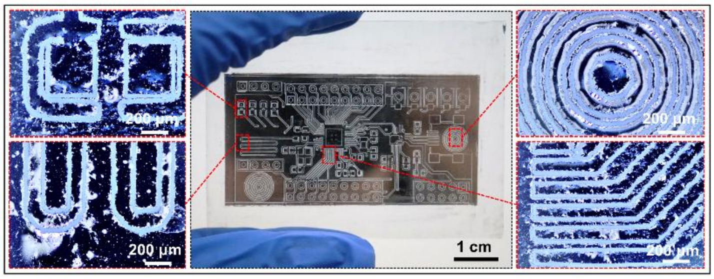

Supplementary Fig. 15 Microscopic detail images of complex semi-liquid metal patterns on glass plates.

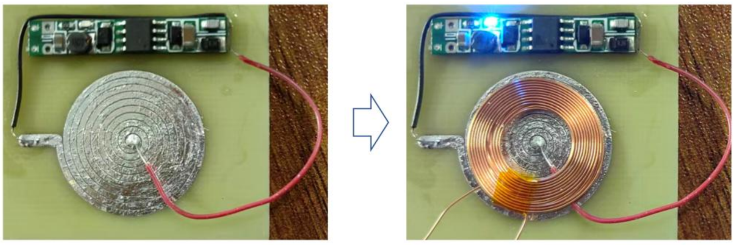

Supplementary Fig. 16 The wireless charging coil.

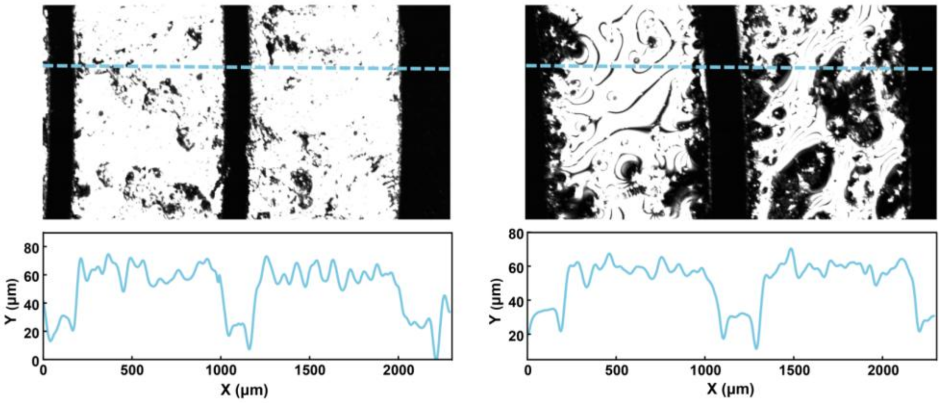

Supplementary Fig. 17 The cross-sectional curve of the two different spaced coils wires (3 biological replicates).

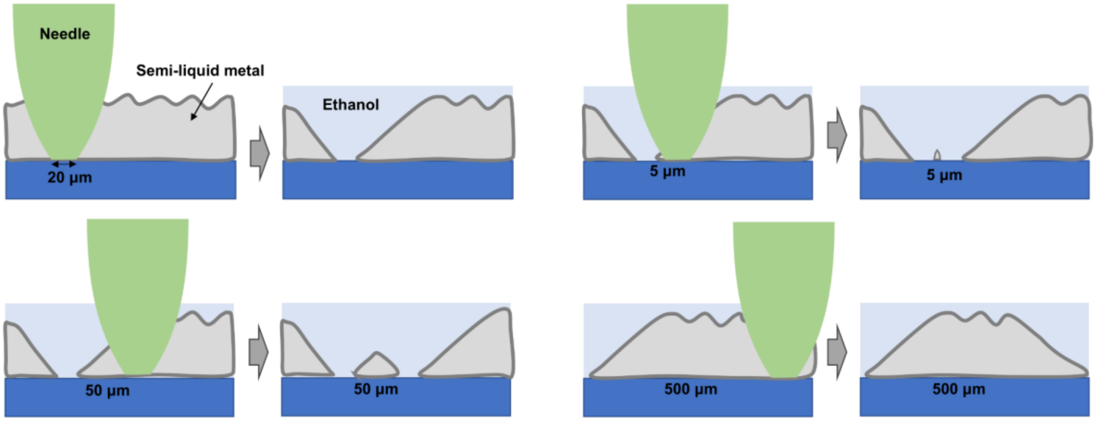

Supplementary Fig. 18 Schematic diagram of semi-liquid metal wire height decreasing with reducing wire width caused by arc-shaped needle tip.

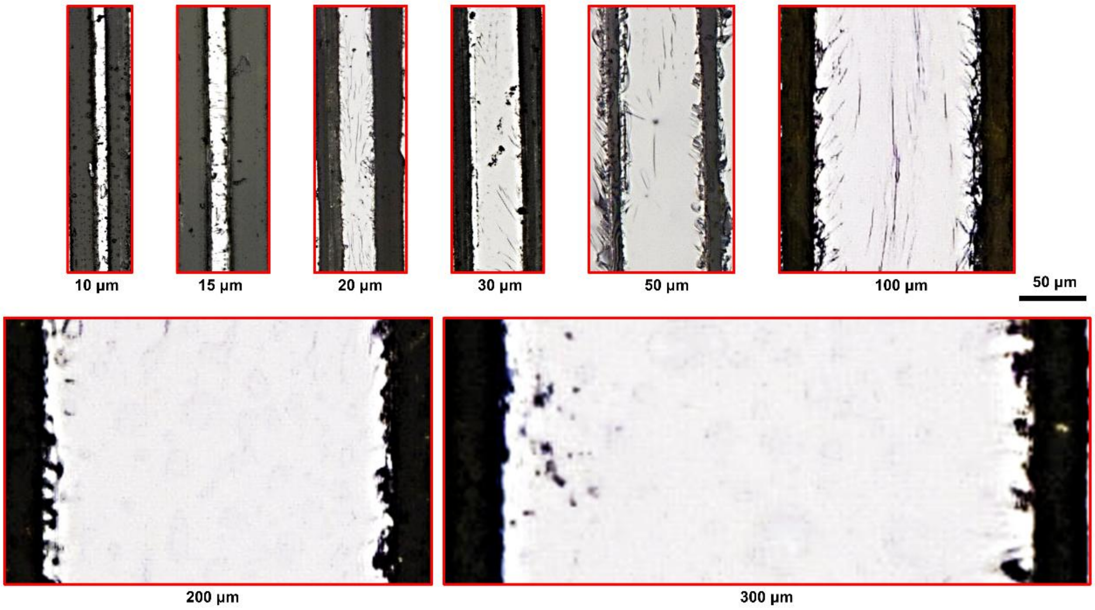

Supplementary Fig. 19 Micrographs of semi-liquid metal wires with different widths (3 biological replicates).

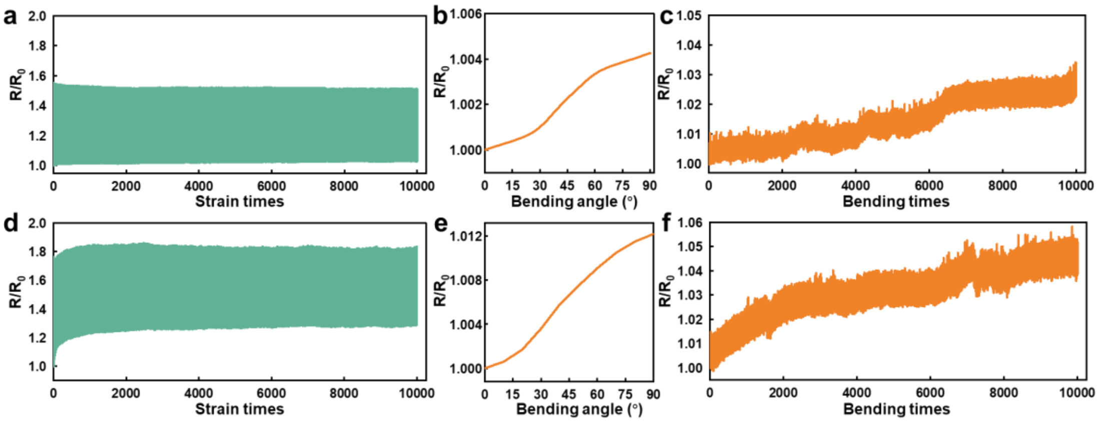

Supplementary Fig. 20 a Resistance variation of semi-liquid metal wire (width of 2 mm) during 10,000 stretching cycles. **b** Relationship between resistance variation and bending angle during semi-liquid metal wire (width of 2 mm) bending. **c** Resistance variation of semi-liquid metal wire (width of 2 mm) during 10,000 bending cycles. **d** Resistance variation of semi-liquid metal wire (width of 0.5 mm) during 10,000 stretching cycles. **e** Relationship between resistance variation and bending angle during semi-liquid metal wire (width of 0.5 mm) bending. **f** Resistance variation of semi-liquid metal wire (width of 0.5 mm) during 10,000 bending cycles.

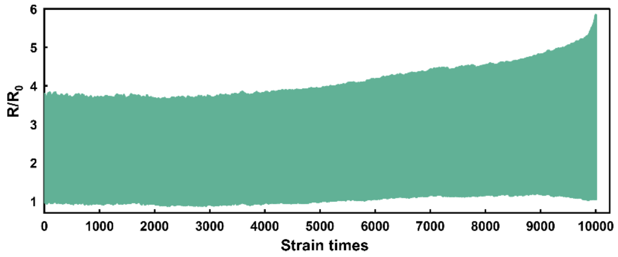

Supplementary Fig. 21 Resistance variation of semi-liquid metal wire under 500% strain during 10,000 stretching cycles.

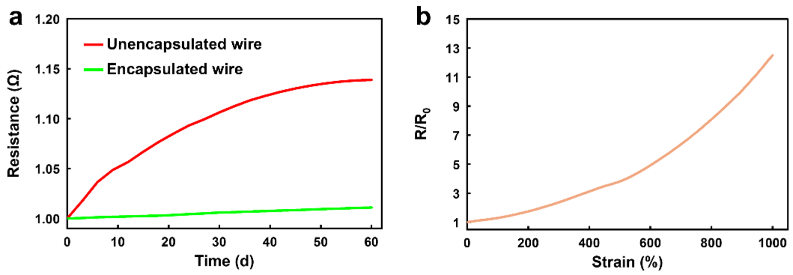

Supplementary Fig. 22 Long-term resistance stability of semi-liquid metal wires. a Resistance-time variation curves of encapsulated and unencapsulated semi-liquid metal wires. **b** Resistance-elongation variation curve of the unencapsulated semi-liquid metal wire after two months of storage.

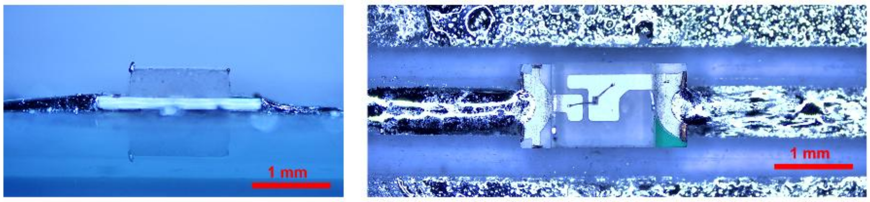

Supplementary Fig. 23 Photographs of wetting soldering between LED pins and semi-liquid metal wires.

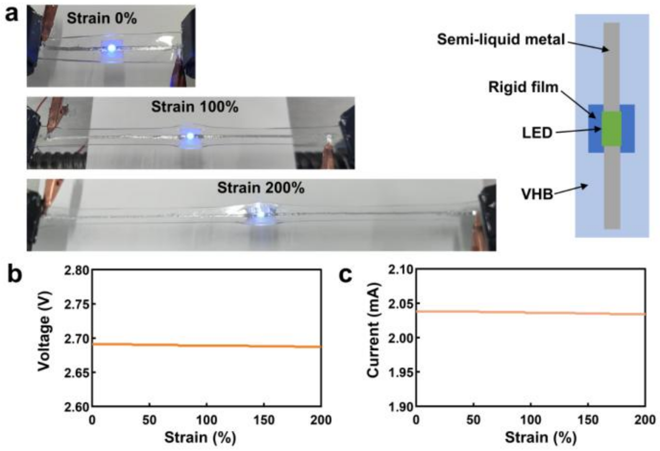

Supplementary Fig. 24 a LED on the VHB tape of composite rigid film under different stretching states. **b** The voltage across the LEDs during 200% stretching process. **c** The current across the LEDs during 200% stretching process.

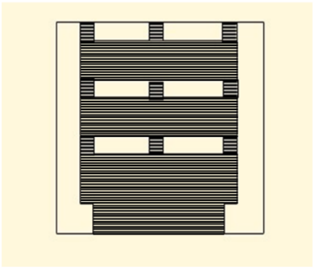

Supplementary Fig. 25 Needle tip moving paths for fabricating LED arrays on PDMS substrates.

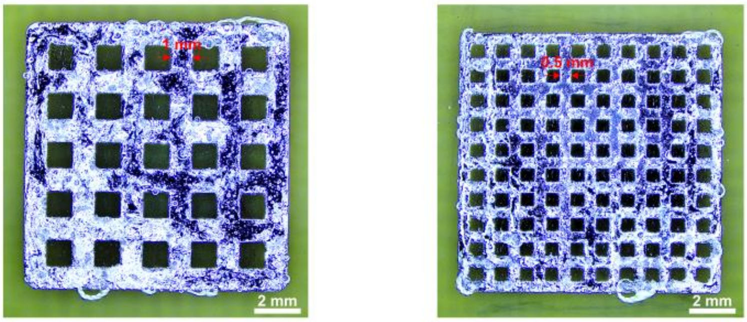

Supplementary Fig. 26 Mesh circuits with two mesh sizes.

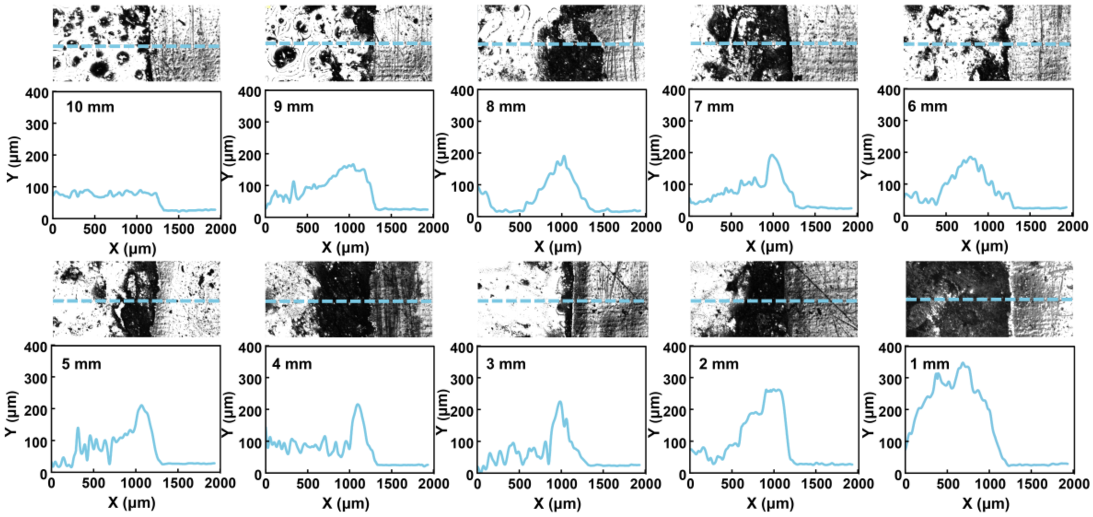

Supplementary Fig. 27 The contour curve of the semi-liquid metal as the line width was reduced from 1 cm to 1 mm (3 biological replicates).

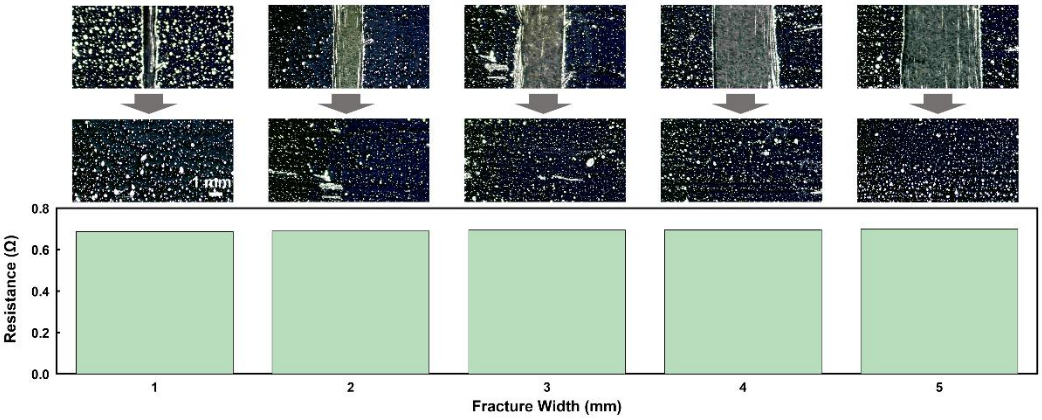

Supplementary Fig. 28 Photographs of scratches with different widths before and after scraper repair, and resistance changes.

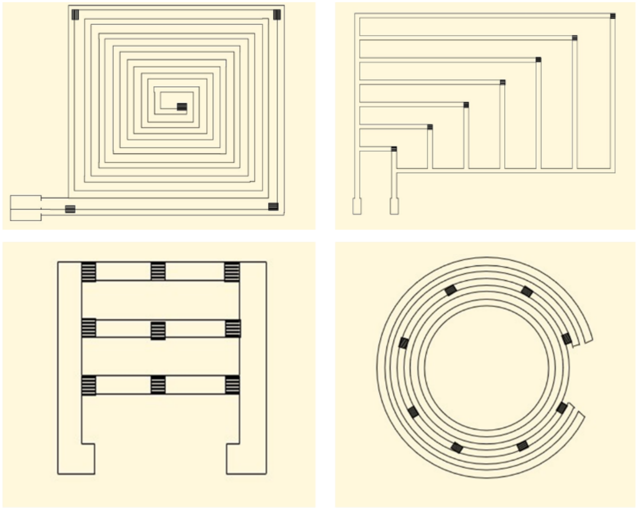

Supplementary Fig. 29 Needle tip moving paths for four circuit patterns in repeated fabrication.

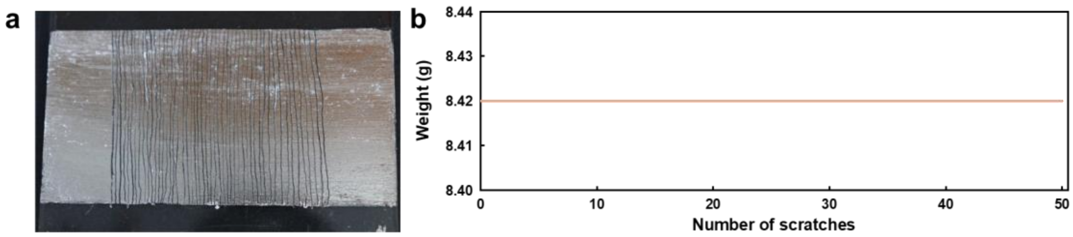

Supplementary Fig. 30 a Photographs of the circuit after 50 repeated engraving cycles. **b** Mass variation curve of the semi-liquid metal during the entire engraving process.

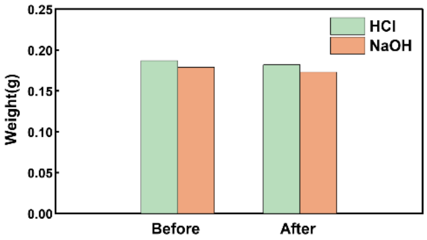

Supplementary Fig. 31 Mass change of semi-liquid metal before and after recovery with two solutions.

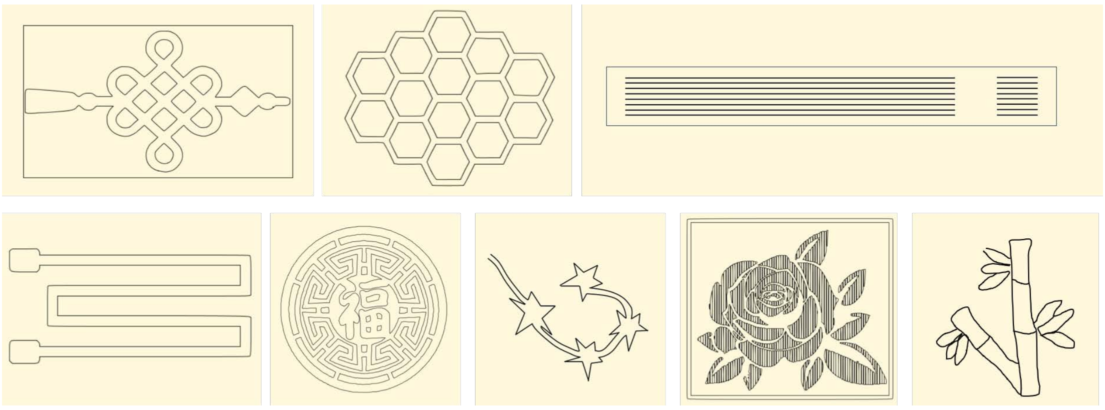

Supplementary Fig. 32 The needle's movement path for 8 substrates.

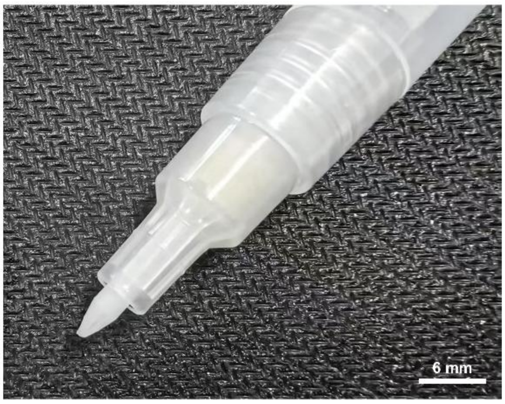

Supplementary Fig. 33 Photo of the alcohol-dipped marker pen

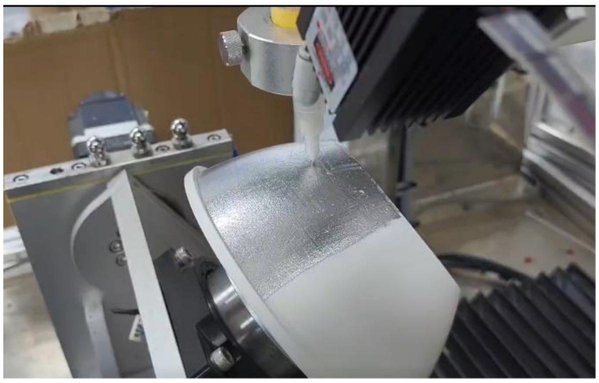

Supplementary Fig. 34 Photo of the 5-axis moving platform

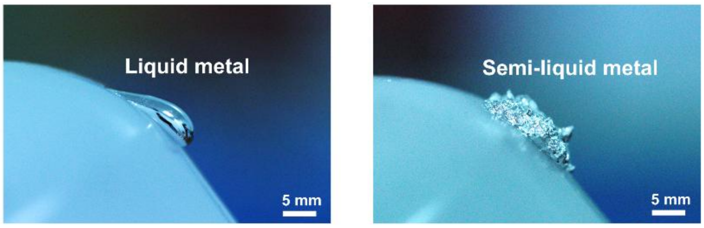

Supplementary Fig. 35 Photographs of liquid metal and semi-liquid metal coated on curved surfaces.

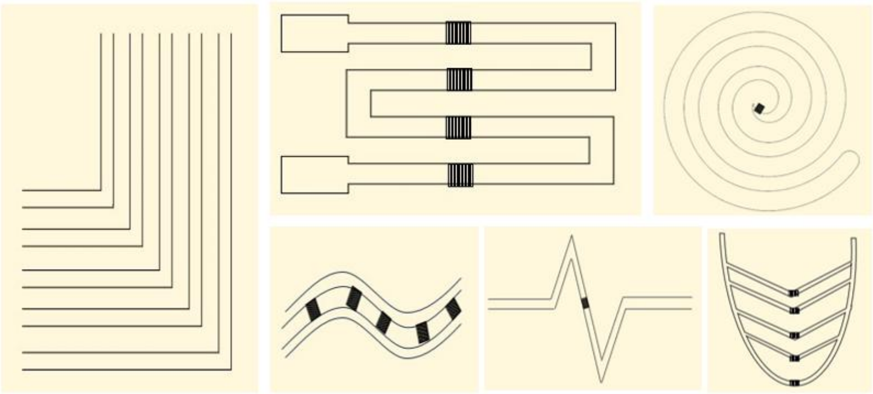

Supplementary Fig. 36 The movement path of the alcohol pen on the curved surface of the 3D-printed models, pepper, egg, and mouse.

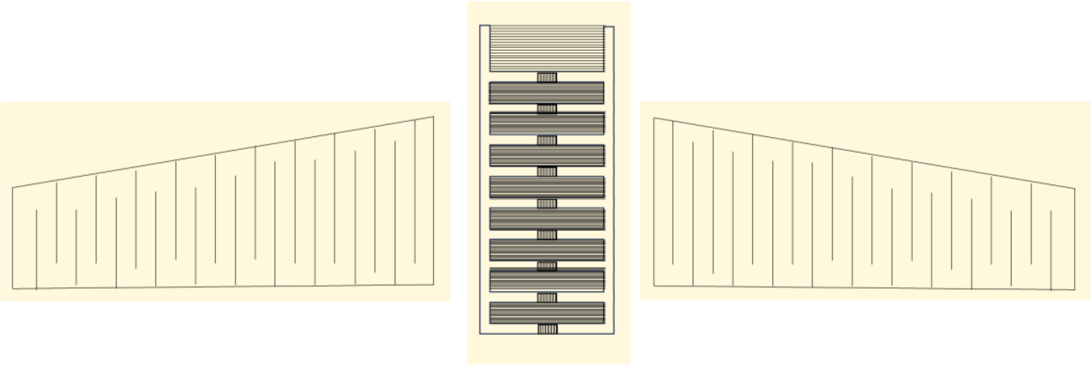

Supplementary Fig. 37 The movement path of the alcohol pen on the curved surface of the fuselage.

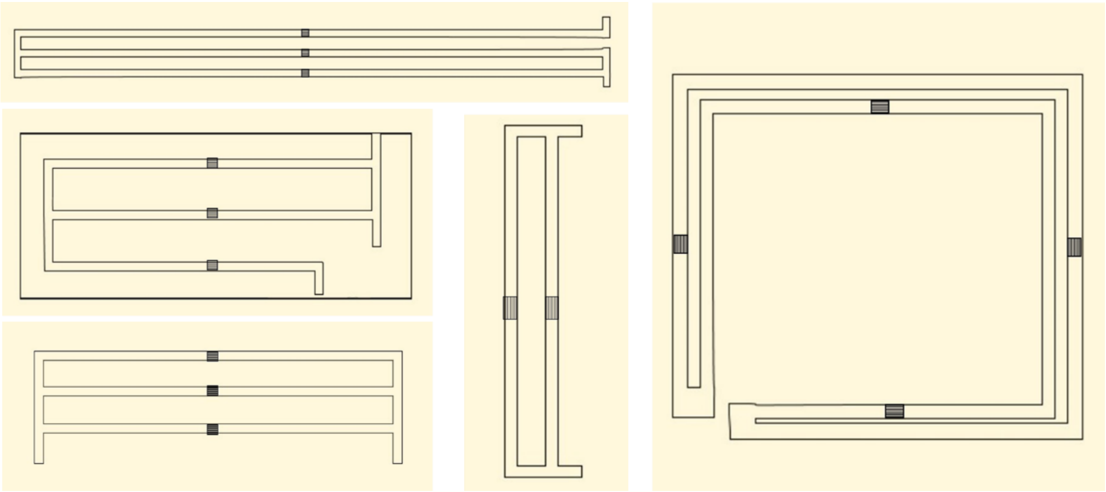

Supplementary Fig. 38 The movement paths of the alcohol pen on the surfaces of three parts of the house model.

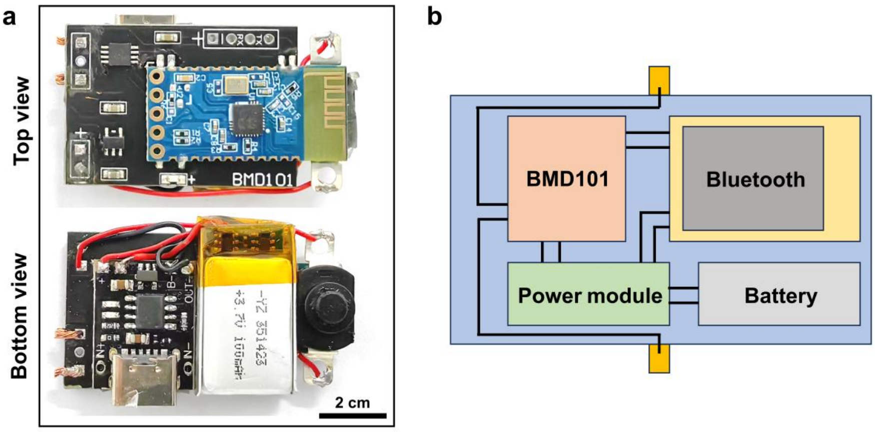

Supplementary Fig. 39 ECG signal acquisition circuit. a Physical image of the circuit. **b** Schematic diagram of the circuit system

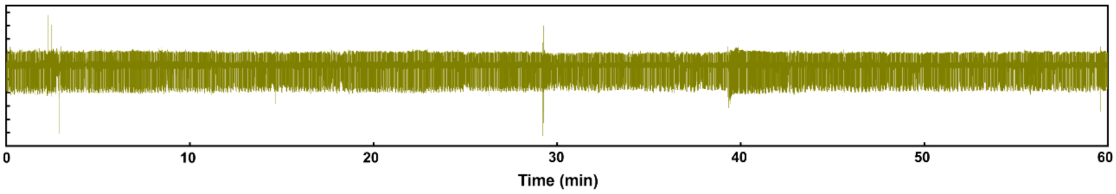

Supplementary Fig. 40 1h ECG waveform.

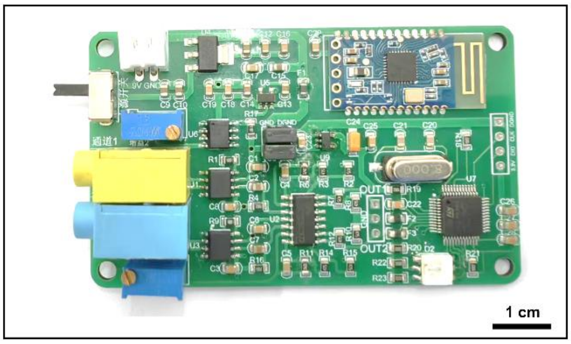

Supplementary Fig. 41 photo of EMG signal acquisition circuit

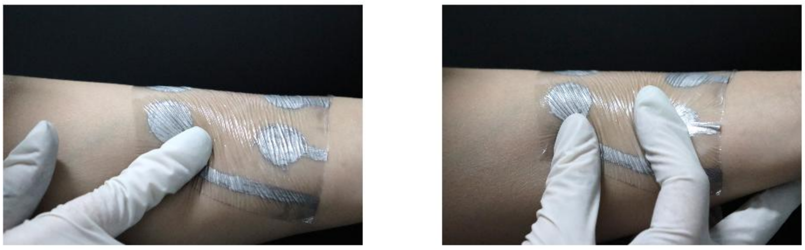

Supplementary Fig. 42 Stable adhesion of EMG electrodes to skin.

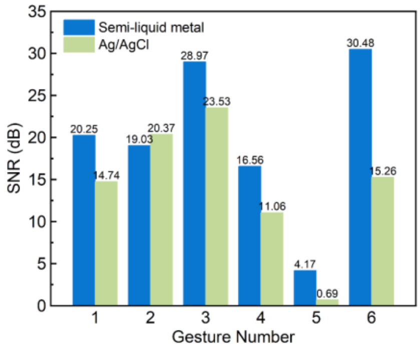

Supplementary Fig. 43 Signal to noise ratio for the semi-liquid metal electrodes and traditional Ag/AgCl electrodes.

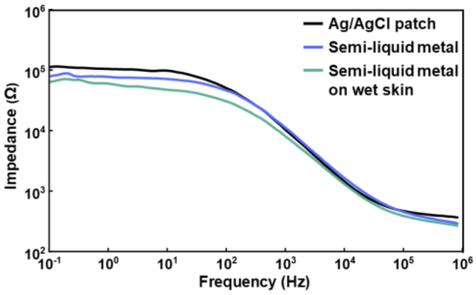

Supplementary Fig. 44 Skin-electrode interface impedance of the semi-liquid metal electrodes and traditional Ag/AgCl electrodes.

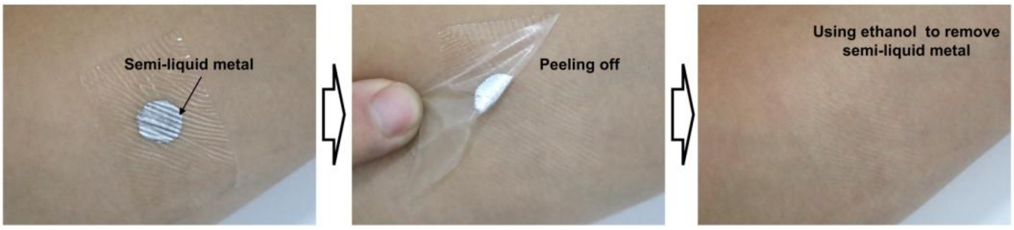

Supplementary Fig. 45 Removal of semi-liquid metal residues after removing the semi-liquid metal electrodes.

Supplementary Table S1 Comparative framework across six key dimensions: manufacturing cost, recyclability, conductivity loss, material loss, resolution, and printability on diverse substrates.

	Manufacture cost	Recyclable	Conductivity (S/m)	Material loss	Resolution(μm)	Printability on various substrates
**This work**	Low	√	9×10^6	×	High (5)	√
**Ref.1**	Low	×	2.9×10^5-1.2×10^6	×	Low (500)	√
**Ref.2**	High	×	3.4×10^6	√	High (20)	×
**Ref.3**	High	×	3×10^6	√	High(20)	×
**Ref.4**	High	√	3.4×10^6	√	High(0.18）	×
**Ref.5**	High	√	3.4×10^6	×	High(5)	√
**Ref.6**	Low	×	1.5×10^6	×	High(50)	√
**Ref.7**	High	√	2.06×10^6	×	High (25)	×
**Ref.8**	Low	√	3.4×10^6	√	Low (100)	×
**Ref.9**	High	×	3.4×10^6	√	350	×
**Ref.10**	High	×	5.65×10^5	√	High(4.5)	×
**Ref.11**	High	×	3.4×10^6	√	Low (150)	×
**Ref.12**	High	×	3.4×10^6	×	Low (200)	×
**Ref.13**	High	√	3.4×10^6	×	Low(200)	×
**Ref.14**	High	×	3.4-6.73×10^6	√	Low (1.3)	×
**Ref.15**	Low	×	4.15×10^4	√	Low (100)	×
**Ref.16**	High	√	3.4×10^6	×	High (1.9)	√
**Ref.17**	High	×	$\sim10^6$	√	Low (138)	×
**Ref.18**	High	√	7.7×10^5	√	High(37)	×
**Ref.19**	High	×	3.4×10^6	√	Low	×
**Ref.20**	Low	√	-	×	Low (250)	×

[1] Rahim, MA. et al. Polyphenol-induced adhesive liquid metal inks for substrate-independent direct pen writing. *Adv. Funct. Mater.* **31**, 2007336 (2021).

[2] Park, C.W. et al. Photolithography-based patterning of liquid metal interconnects for monolithically integrated stretchable circuits. *ACS Appl. Mater. Interfaces.* **8**, 15459-15465 (2016).

[3] Wu, D. et al. Fast and facile liquid metal printing via projection lithography for highly stretchable electronic circuits. *Adv. Mater.* **36**, 2307632 (2024).

[4] Kim, M-g., Brown, D.K., Brand O. Nanofabrication for all-soft and high-density electronic devices based on liquid metal. *Nat. Commun.* **11**, 1002 (2020).

[5] Yun, I. et al. Transferable transparent electrodes of liquid metals for bifacial perovskite solar cells and heaters. *Nano Energy.* **93**, 106857 (2022).

[6] Lee, G-H.et al. Rapid meniscus-guided printing of stable semi-solid-state liquid metal microgranular-particle for soft electronics. *Nat. Commun.* **13**, 2643 (2022).

[7] Liu, S., Shah, D.S., Kramer-Bottiglio, R. Highly stretchable multilayer electronic circuits using biphasic gallium-indium. *Nat. Mater.* **20**, 851-858 (2021).

[8] Park, J-E., Kang, H.S., Koo, M., Park, C. Autonomous surface reconciliation of a liquid-metal conductor micropatterned on a deformable hydrogel. *Adv. Mater.* **32**, 2002178 (2020).

[9] Wei, Y. et al. Liquid metal grid patterned thin film devices toward absorption-dominant and strain-tunable electromagnetic interference shielding. *Nano-Micro Lett.* **16**, 248 (2024).

[10] Minsik K. et al. Ambient printing of native oxides for ultrathin transparent flexible circuit boards. *Science.* **385**, 731-737(2024).

[11] Bhuyan, P. et al. Multifunctional ultrastretchable and ultrasoft electronics enabled by uncrosslinked polysiloxane elastomers patterned with rheologically modified liquid metal electrodes: beyond current soft and stretchable electronics. *Chem. Eng. J.* **453** ,1385-8947 (2022).

[12] Lu, T., Finkenauer, L., Wissman, J., Majidi, C. Rapid prototyping for soft-matter electronics. *Adv. Funct. Mater.* **24**, 3351-3356 (2014).

[13] Li, N., et al. Fingertip-inspired spatially anisotropic inductive liquid metal sensors with ultra-wide range, high linearity and exceptional stability. *Adv. Mater.* **37**, 2419524 (2025).

[14] Chen, X. et al. Compression-durable soft electronic circuits enabled by embedding self-healing biphasic liquid-solid metal into microstructured elastomeric channels. *Adv. Mater.* **37,** 2420469 (2025).

[15] Wang, Z. et al. Three dimensional core-shell structured liquid metal/elastomer composite via coaxial direct ink writing for electromagnetic interference shielding. *Compos. Part. A-Appl.* S.**136**, (2020).

[16] Park, Y-G., An, H.S., Kim, J-Y., Park, J-U. High-resolution, reconfigurable printing of liquid metals with three-dimensional structures. *Sci. Adv.***5**, eaaw2844(2019).

[17] Li, S., Zhao, H., Xu, H., Lu, H., Luo, P., Zhou, T. Ultra-flexible stretchable liquid metal circuits with antimicrobial properties through selective laser activation for health monitoring. *Chem. Eng. J.***482** , 149173 (2024).

[18]Liu, S., Kim, S.Y., Henry, K.E., Shah, D.S., Kramer-Bottiglio, R. Printed and Laser-Activated Liquid Metal-Elastomer Conductors Enabled by Ethanol/PDMS/Liquid Metal Double Emulsions. *ACS Appl. Mater. Interfaces.* **13**, 28729-28736 (2021).

[19] Li ,Y., Feng, S., Cao, S., Zhang, J., Kong, D. Printable Liquid Metal Microparticle Ink for Ultrastretchable Electronics. *ACS Appl. Mater. Interfaces.* **12,** 50852-50859 (2020).

[20] L. Teng, S. C. Ye, S. Handschuh-Wang, X. H. Zhou, T. S. Gan, X. C. Zhou. Liquid Metal-Based Transient Circuits for Flexible and Recyclable Electronics.*Adv. Funct. Mater.* **29**, 1808739(2019).
