# Supplementary material for: Non-loss engraved circuit patterning method of semi-liquid metal for precision recyclable multi-substrate circuits
Source: Nat Commun. 2025 Nov 27;16:11690. doi: 10.1038/s41467-025-66815-4 (PMC12748806; doi:10.1038/s41467-025-66815-4)
Supplement: Supplementary file 2 — Description of Additional Supplementary Information [file 41467_2025_66815_MOESM2_ESM.pdf]

Description of Additional Supplementary Information

Supplementary Video 1 Fabrication of liquid metal wire arrays via NECP method.

Supplementary Video 2 The contact angles of alcohol on different substrates.

Supplementary Video 3 Fabrication of the complex circuit via NECP method.

Supplementary Video 4 Non-loss engraving of liquid metal wire.

Supplementary Video 5 NaOH and HCl solution recovery circuit.

Supplementary Video 6 NECP method on the 3D curved surface.
