# Supplementary material for: Non-loss engraved circuit patterning method of semi-liquid metal for precision recyclable multi-substrate circuits
Source: Nat Commun. 2025 Nov 27;16:11690. doi: 10.1038/s41467-025-66815-4 (PMC12748806; doi:10.1038/s41467-025-66815-4)
Supplement: Supplementary file 9 — Reporting Summary [file 41467_2025_66815_MOESM9_ESM.pdf]

nature portfolio

Corresponding author(s): Jing Liu, Rui Guo

Last updated by author(s): Oct 31, 2025

Reporting Summary

Nature Portfolio wishes to improve the reproducibility of the work that we publish. This form provides structure for consistency and transparency in reporting. For further information on Nature Portfolio policies, see our Editorial Policies and the Editorial Policy Checklist.

Statistics

For all statistical analyses, confirm that the following items are present in the figure legend, table legend, main text, or Methods section.

n/a	Confirmed	
☐	☒	The exact sample size (*n*) for each experimental group/condition, given as a discrete number and unit of measurement
☐	☒	A statement on whether measurements were taken from distinct samples or whether the same sample was measured repeatedly
☒	☐	The statistical test(s) used AND whether they are one- or two-sided<br>*Only common tests should be described solely by name; describe more complex techniques in the Methods section.*
☒	☐	A description of all covariates tested
☒	☐	A description of any assumptions or corrections, such as tests of normality and adjustment for multiple comparisons
☐	☒	A full description of the statistical parameters including central tendency (e.g. means) or other basic estimates (e.g. regression coefficient) AND variation (e.g. standard deviation) or associated estimates of uncertainty (e.g. confidence intervals)
☒	☐	For null hypothesis testing, the test statistic (e.g. *F*, *t*, *r*) with confidence intervals, effect sizes, degrees of freedom and *P* value noted<br>*Give P values as exact values whenever suitable.*
☒	☐	For Bayesian analysis, information on the choice of priors and Markov chain Monte Carlo settings
☒	☐	For hierarchical and complex designs, identification of the appropriate level for tests and full reporting of outcomes
☒	☐	Estimates of effect sizes (e.g. Cohen's *d*, Pearson's *r*), indicating how they were calculated

Our web collection on statistics for biologists contains articles on many of the points above.

Software and code

Policy information about availability of computer code

Data collection	The electrical conductivity of semi-liquid metal with varying doping ratios was determined via the standard four-point probe method. The resistances of semi-liquid metal wire samples with different widths were measured by a digital multimeter (Keithley 2002, Tektronix, Inc.). A four-terminal method was employed to avoid the influence of contact resistance. The semi-liquid metal wires (with a width of 1 mm and a length of 2 cm) on the VHB substrate and the PI substrate were fixed onto a dynamic mechanical test system (HC-01, Dongtai Suheng Transmission Technology Co., Ltd.), and a multimeter (U1251B, Agilent Technologies, Inc.) was used to measure the resistance changes of the two types of wires during the stretching process (on the VHB substrate) and the bending process (on the PI substrate).An infrared camera (FOTRIC 220s, Fotric Smart Technology Co., Ltd.) was used to measure the temperature distribution of the heating circuit on wings.<br>A contact angle measuring instrument (SDC-200, Shengding Precision Instrument Co., Ltd) was used to measure the contact angles of liquid metal droplets on various substrates. To characterize the adhesion between semi-liquid metal and substrate, the critical slip angles were tested on a platform with an adjustable incline angle, and recorded using a camera (EOS 200D II, Canon Inc.). 3D images and cross-sections were obtained using a laser confocal microscope (Olympus, LEXT OLS 4000). The valence states of elements were characterized by XPS (ESCALAB 250Xi, Thermo Fisher Scientific, Oxford, UK).<br>An analog front-end amplifier (BMD101, NeuroSky Electronic Technology Co., Ltd) was used to collect ECG signals. An analog front-end amplifier (ADS1298, Texas Instruments, Inc., Dallas, TX, USA) was used to collect EMG signals.
Data analysis	The data was analyzed with MATLAB R2022b and Origin 2022

For manuscripts utilizing custom algorithms or software that are central to the research but not yet described in published literature, software must be made available to editors and reviewers. We strongly encourage code deposition in a community repository (e.g. GitHub). See the Nature Portfolio guidelines for submitting code & software for further information.

Data

Policy information about availability of data

All manuscripts must include a data availability statement. This statement should provide the following information, where applicable:

- Accession codes, unique identifiers, or web links for publicly available datasets
- A description of any restrictions on data availability
- For clinical datasets or third party data, please ensure that the statement adheres to our policy

The data generated in this study are provided in the Supplementary Information/Source Data file.

Research involving human participants, their data, or biological material

Policy information about studies with human participants or human data. See also policy information about sex, gender (identity/presentation), and sexual orientation and race, ethnicity and racism.

Reporting on sex and gender	One participant whose sex (and gender) is male. Sex (or gender) was not considered in the study design since it is a proof of concept designed to demonstrate that liquid metal electrodes can collect electrophysiological signals from the human body surface.
Reporting on race, ethnicity, or other socially relevant groupings	Han Chinese male
Population characteristics	The participant is from China and aged 29.
Recruitment	The participant is one of the authors of this article.
Ethics oversight	All procedures involving the attachment of electrodes to human skin comply with ethical guidelines, which have been approved by Tianjin University (approval number: TJUE2025-H-S-063).

Note that full information on the approval of the study protocol must also be provided in the manuscript.

Field-specific reporting

Please select the one below that is the best fit for your research. If you are not sure, read the appropriate sections before making your selection.

☒ Life sciences ☐ Behavioural & social sciences ☐ Ecological, evolutionary & environmental sciences

For a reference copy of the document with all sections, see nature.com/documents/nr-reporting-summary-flat.pdf

Life sciences study design

All studies must disclose on these points even when the disclosure is negative.

Sample size	One participant whose sex (and gender) is male. Sex (or gender) was not considered in the study design since it is a proof of concept designed to demonstrate that liquid metal electrodes can collect electrophysiological signals from the human body surface.
Data exclusions	No data was excluded.
Replication	All EMG and ECG measurement were repeated for 3 times (1h per time) to verify its reproducibility.
Randomization	These samples were not collected randomly, as this is a proof-of-concept study to verify the effectiveness of the electrodes.
Blinding	Blinding was not required in this study, as its purpose is to provide a proof of concept for verifying the effectiveness of the electrodes.

Reporting for specific materials, systems and methods

We require information from authors about some types of materials, experimental systems and methods used in many studies. Here, indicate whether each material, system or method listed is relevant to your study. If you are not sure if a list item applies to your research, read the appropriate section before selecting a response.

Materials & experimental systems

n/a	Involved in the study
☒	☐ Antibodies
☒	☐ Eukaryotic cell lines
☒	☐ Palaeontology and archaeology
☒	☐ Animals and other organisms
☒	☐ Clinical data
☒	☐ Dual use research of concern
☒	☐ Plants

Methods

n/a	Involved in the study
☒	☐ ChIP-seq
☒	☐ Flow cytometry
☒	☐ MRI-based neuroimaging

Plants

Seed stocks	not applicable
Novel plant genotypes	not applicable
Authentication	not applicable
